# Hereditary neuropathy with liability to pressure palsies with recurrent facial paralysis as main clinical manifestation in a Chinese patient: A case report

**DOI:** 10.1097/MD.0000000000043192

**Published:** 2025-07-04

**Authors:** Min Zhao, Qicui Du, Yanan Ding, Weifei Wang

**Affiliations:** a Department of Neurology, Liaocheng People’s Hospital, Liaocheng, Shandong Province, China; b Department of Stomatology, Liaocheng People’s Hospital, Liaocheng, Shandong Province, China.

**Keywords:** facial paralysis, HNPP, neuroelectrophysiological characteristics, PMP22 gene

## Abstract

**Rationale:**

Hereditary neuropathy with liability to pressure palsies (HNPP) is a rare autosomal dominant genetic disorder characterized by recurrent, brief, and painless nerve paralysis after mild compression. Common peroneal nerve, ulnar nerve, and median nerve are most often involved, but cranial nerve involvement is rare. However, we found a case of HNPP with recurrent facial paralysis as main clinical manifestation.

**Patient concerns:**

A 31-year-old male experienced 3 episodes of left peripheral facial paralysis over the past 4 years. After hospitalization, we found that the patient had weakened tendon reflex of the limbs and talipes cavus, which highly suggests chronic peripheral neuropathy.

**Diagnoses:**

The electromyography examination was performed. Motor nerve conduction detection found conduction block of the right common peroneal nerve at the fibular head; sensory nerve conduction detection showed a general slowdown in sensory nerve conduction velocity. The gene sequencing results showed that the patient carries heterozygous deletion of PMP22 gene (exon 1–5 deletion). Therefore, the final diagnosis was HNPP.

**Interventions:**

The patient was treated with oral prednisone and B vitamins.

**Outcomes:**

The patient was given 20 mg of prednisone once a day for 1 week, and then reduced by 5 mg every 3 days until discontinuation, supplemented with B vitamins. The patient gradually improved and fully recovered to normal after 40 days.

**Lessons:**

Facial nerve paralysis is an atypical clinical presentation of HNPP. This is the first report of HNPP in a Han Chinese population with recurrent facial nerve paralysis as the main symptom. This case has further enriched the clinical spectrum of HNPP, which is worthy of reference for neurologists.

## 1. Introduction

Hereditary neuropathy with liability to pressure palsies (HNPP) is a rare autosomal dominant genetic disorder, characterized by recurrent painless single or multiple nerve paralysis after peripheral nerve tension or compression.^[1]^ In clinical practice, the clinical manifestations of some patients are mild or atypical, which can easily lead to missed diagnosis or misdiagnosis. The most commonly affected nerves are the peroneal nerve, ulnar nerve, median nerve, and brachial plexus nerve,^[2–4]^ while reports of cranial nerve involvement are rare.^[5–7]^ We report a genetically confirmed case of HNPP with recurrent peripheral facial paralysis as the main manifestation, in order to improve the understanding and diagnostic level of clinical physicians about this disease.

## 2. Case report

The patient, a 31-year-old male, had recurrent episodes of left peripheral facial paralysis in the past 4 years. The following is the developments of the patient. In May 2020, the patient experienced weak closure of the left eyelid and left-side labial commissure deviation, which was diagnosed as facial neuritis, and completely recovered after symptomatic treatments for about half a month. In August 2022, he developed right common peroneal nerve paralysis after crossing her legs for a long time. He was treated with prednisone and vitamin B12, and the symptoms were completely relieved 2 months later. In May 2023, the patient again developed mild left peripheral facial palsy, which recovered spontaneously after 3 weeks, although no special treatment was given. On March 4, 2024, the patient experienced a recurrence of left eyelid closure weakness and mouth angle deviation without any obvious cause. Up to now, the patient has experienced 3 episodes of left peripheral facial paralysis, none of which were accompanied by taste disorders or auditory allergies. On neurological examination, he exhibited left peripheral facial palsy, weakened tendon reflexes in the limbs, and high foot arches. The remaining exam revealed no abnormalities, including signs of other cranial neuropathies or limb weakness. No vesicles in the external ear were observed and otoscopy was unremarkable.

Brain and facial nerve magnetic resonance imaging were normal. No laboratory sign of inflammation was recorded. Serology of Lyme disease, syphilis, HIV infection, and infection with other rarer neurotropic viruses were negative. The electromyography examination was performed. Motor nerve conduction detection found that the latency period of the bilateral median nerve was prolonged, and the conduction velocity of the bilateral ulnar nerve and common peroneal nerve was slowed down, which was obvious in prone compression areas. There was conduction block of the right common peroneal nerve at the fibular head (Table 1). Sensory nerve conduction detection showed a general slowdown in sensory nerve conduction velocity, and even some nerves did not elicit waveforms (Table 2).

**Table 1:** Results of the motor nerve conduction detection.

Project	Stimulus points	Normal values	Left side	Right side
DML (ms)				
Median nerve	Wrist	≤3.8	4.7	4.4
Ulnar nerve	Wrist	≤3.0	2.8	2.8
Peroneal nerve	Ankle	≤4.7	4.5	4.6
Tibial nerve	Ankle	≤5.1	4.3	3.9
CMAP (mv)				
Median nerve	Wrist	≥9	10.8	11.4
	Elbow	≥7	9.2	10.5
Ulnar nerve	Wrist	≥8	11.3	9.2
	Below the elbow	≥8	11.1	8.9
	Above the elbow	≥7	10.5	8.6
Peroneal nerve	Ankle	≥3.6	4.3	4.1
	Below the fibular head	≥3.4	3.6	3.5
	Above the fibular head	≥3.4	3.1	1.3
Tibial nerve	Ankle	≥4	4.7	4.6
	Popliteal fossa	≥4	4.6	4.2
MNCV (m/s)				
Median nerve	Elbow–wrist	≥50	54.6	56.3
Ulnar nerve	Below the elbow–wrist	≥50	55.2	52.1
	Above–below the elbow	≥50	24.7	29.2
Peroneal nerve	Below the fibular head–ankle	≥40	41.5	37.4
	Above–below the fibular head	≥40	34.2	29.3
Tibial nerve	Popliteal fossa–ankle	≥40	41.7	43.6

CMAP = compound muscle action potential, DML = distalmotorlatency, MNCV = motor nerve conduction velocity.

**Table 2:** Results of the sensory nerve conduction detection.

Project	Stimulus points	Normal values	Left side	Right side
SNAP (μV)				
Median nerve	Finger I	≥21	40.1	34.8
Ulnar nerve	Finger V	≥7.1	17.9	17.8
Tibial nerve	Toe I	≥0.7	–	–
Superficial peroneal nerve	Lateral crural region	≥0.7	0.6	–
Sural nerve	Lateral malleolus	≥3.2	11.1	8.1
SNCV (m/s)				
Median nerve	Finger I	≥45	37	36
Ulnar nerve	Finger V	≥45	29	39
Tibial nerve	Toe I	≥35	–	–
Superficial peroneal nerve	Lateral crural region	≥40	36	–
Sural nerve	Lateral malleolus	≥40	42	40

– = unelicited waveform, SNAP = sensory nerve action potential, SNCV = sensory nerve conduction velocity.

**Figure 1.**
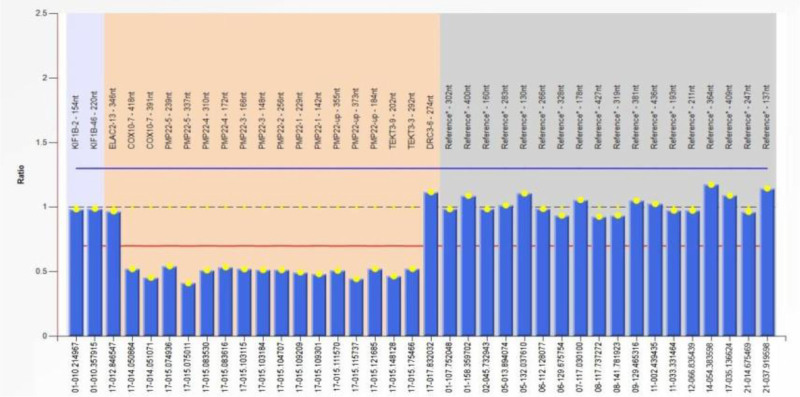
The gene sequencing results showed that the patient carries heterozygous deletion of PMP22 gene (exon 1–5 deletion). Note: A fluorescence signal intensity between 0.75 and 1.25 is normal, <0.75 indicates absence, and >1.25 indicates repetition. PMP22 = peripheral myelin protein 22.

Based on the patient’s clinical and neuroelectrophysiological characteristics, it is highly suspected to be HNPP. Further genetic testing showed that there was a large heterozygous deletion mutation in the exon region of the Peripheral myelin protein-22 (PMP22) gene of the patient (Fig. 1), thus the final diagnosis was HNPP. Unfortunately, the patient’s parents refused the relevant genetic testing. The patient was given 20 mg of prednisone once a day for 1 week, and then reduced by 5 mg every 3 days until discontinuation, supplemented with B vitamins. The patient gradually improved and was followed up until April 15, 2024, when the patient fully recovered to normal.

## 3. Discussion

HNPP is a rare autosomal dominant hereditary demyelinating peripheral neuropathy with a prevalence rate of approximately (8.4–16) per 100,000 people.^[8]^ The pathogenesis of this disease is the deletion or point mutation of chromosome 17p11.2 of the PMP22 gene, which leads to a decrease in the expression of PMP22 in the myelinated fibers of the peripheral nerves.^[9]^ The typical clinical manifestation is recurrent, brief, and painless paralysis of nerves that are prone to compression after slight compression, with the common peroneal nerve, ulnar nerve, and median nerve being the most common, followed by brachial plexus and radial nerve, and cranial nerve being rare.^[10]^ The incidence rate of this disease may be underestimated due to the heterogeneity of its clinical manifestations and the fact that patients often have a benign course, or even no clinical symptoms.

The prominent feature of the patient in this case is repeated left peripheral facial paralysis, with no brainstem abnormalities observed on cranial magnetic resonance imaging. Facial nerve conduction examination shows prolonged latency, indicating left lateral nerve paralysis. The facial nerve is the longest cranial nerve in the human body that passes through bony ducts and is also the most prone to paralysis. The most common cause of facial nerve paralysis is facial neuritis.^[11]^ In 1998, Poloni TE et al reported a case of HNPP with recurrent peripheral facial paralysis as the sole clinical manifestation,^[12]^ but there have been few reports since then. This case is the first reported case of HNPP in a Han Chinese population with recurrent facial nerve paralysis as the main manifestation. The patient has experienced multiple episodes of peripheral facial paralysis without accompanying taste disorders or auditory allergies, indicating that the facial nerve was damaged after branching out of the stapedius nerve. The pathogenesis of HNPP remains unclear, and it is widely accepted that reduced expression of PMP22 makes nerves more susceptible to conduction block when subjected to pressure or tension.^[1]^ Facial nerves that have myelin sheath lesions and traverse bony canals may be more prone to slight pressure, which can result in facial paralysis.

Given the high clinical heterogeneity of HNPP and the fact that a considerable number of patients have no family history, neurophysiological examination, as a fast and accurate diagnostic method, can detect a wide range of subclinical neurological damage, which is crucial for the clear diagnosis of the disease.^[13]^ Through detailed physical examination, we found that the patient had weakened tendon reflex of the limbs and talipes cavus, which highly suggests chronic peripheral neuropathy. Therefore, we conducted neurophysiological examinations, providing a basis for further genetic testing. HNPP patients can experience widespread slowing of sensory nerve conduction velocity in the early stages of the disease, and the number of affected peripheral nerves exceeds the range of clinical symptoms.^[14]^ Although the clinical manifestations are mainly motor symptoms, the degree of slowing of motor nerve conduction velocity is relatively mild. The incidence of prolonged distal motor latency is high, nerve conduction block may occur in areas prone to compression.^[15]^ Therefore, when a patient presents with mild compression and then experiences nerve paralysis, neurophysiological examination should be performed, and the scope of electrophysiological examination should be expanded.^[16]^ In addition to examining clinically affected nerves, motor and sensory conduction tests of the bilateral median nerve and peroneal nerve should also be performed.^[17]^ When HNPP is highly suspected, the patient’s family history should be traced, and relevant gene testing should be performed.

At present, there is no specific treatment method for HNPP.^[18]^ In this case, the patient was treated with B group vitamins and low-dose glucocorticoids, and the symptoms were completely relieved. In the acute phase of HNPP, neither excessive rehabilitation treatment nor nerve decompression surgery is recommended to avoid unnecessary nerve damage.

The specific mechanism by which the patient experienced recurrent facial nerve paralysis in this case is not yet clear. We speculate that during the course of the left facial nerve, it is possible for the surrounding tissues to experience slight compression or tension due to some special external force. This case has further enriched the clinical manifestation spectrum of HNPP, which is worthy of reference for neurologists.

## Author contributions

**Conceptualization:** Min Zhao, Weifei Wang.

**Data curation:** Qicui Du.

**Formal analysis:** Min Zhao, Qicui Du, Yanan Ding.

**Investigation:** Qicui Du, Yanan Ding.

**Writing – original draft:** Min Zhao.

**Writing – review & editing:** Weifei Wang.
